# Farnesylthiosalicylic acid sensitizes hepatocarcinoma cells to artemisinin derivatives

**DOI:** 10.1371/journal.pone.0171840

**Published:** 2017-02-09

**Authors:** Liping Wu, Yilin Pang, Guiqi Qin, Gaina Xi, Shengnan Wu, Xiaoping Wang, Tongsheng Chen

**Affiliations:** 1 MOE Key Laboratory of Laser Life Science & Institute of Laser Life Science, College of Biophotonics, South China Normal University, Guangzhou, Guangdong, PR China; 2 Department of Pain Management, The First Affiliated Hospital of Jinan University, Guangzhou, Guangdong, PR China; University of South Alabama Mitchell Cancer Institute, UNITED STATES

## Abstract

Dihydroartemisinin (DHA) and artesunate (ARS), two artemisinin derivatives, have efficacious anticancer activities against human hepatocarcinoma (HCC) cells. This study aims to study the anticancer action of the combination treatment of DHA/ARS and farnesylthiosalicylic acid (FTS), a Ras inhibitor, in HCC cells (Huh-7 and HepG2 cell lines). FTS pretreatment significantly enhanced DHA/ARS-induced phosphatidylserine (PS) externalization, Bak/Bax activation, mitochondrial membrane depolarization, cytochrome *c* release, and caspase-8 and -9 activations, characteristics of the extrinsic and intrinsic apoptosis. Pretreatment with Z-IETD-FMK (caspase-8 inhibitor) potently prevented the cytotoxicity of the combination treatment of DHA/ARS and FTS, and pretreatment with Z-VAD-FMK (pan-caspase inhibitor) significantly inhibited the loss of ΔΨm induced by DHA/ARS treatment or the combination treatment of DHA/ARS and FTS in HCC cells. Furthermore, silencing Bak/Bax modestly but significantly inhibited the cytotoxicity of the combination treatment of DHA/ARS and FTS. Interestingly, pretreatment with an antioxidant N-Acetyle-Cysteine (NAC) significantly prevented the cytotoxicity of the combination treatment of DHA and FTS instead of the combination treatment of ARS and FTS, suggesting that reactive oxygen species (ROS) played a key role in the anticancer action of the combination treatment of DHA and FTS. Similar to FTS, DHA/ARS also significantly prevented Ras activation. Collectively, our data demonstrate that FTS potently sensitizes Huh-7 and HepG2 cells to artemisinin derivatives via accelerating the extrinsic and intrinsic apoptotic pathways.

## Introduction

Hepatocellular carcinoma (HCC) is the fifth most common cancers and the second most lethal cancer worldwide [1,2]. More than 700,000 cases of HCC are diagnosed and as many as 500,000 people die from HCC annually [3,4]. Several approaches are available for HCC therapy including surgical resection, liver transplantation, chemotherapy and radiotherapy [3–7]. Surgical resection and liver transplantation are two main curative treatments for patients with early HCC [2]. In fact, only a minority of the patients can be offered a curative treatment because most patients are often diagnosed at advanced stages of HCC [5]. High resistance of HCC to available chemotherapeutic agents and the low tolerance of the liver to irradiation result in the limitation of chemotherapy and radiotherapy [1]. Therefore, discovery and development of innovative anti-HCC agents with lower host toxicity has turned to natural sources and their combined treatment with other drugs [8–14].

Dihydroartemisinin (DHA) and artesunate (ARS), two artemisinin derivatives (ARTs), exhibit potent anticancer activity in many cancer cell lines [12,15–17] and synergistic anticancer effect with other drugs [10,11,18]. It was reported that the anticancer activity of tumor necrosis factor-related apoptosis inducing ligand (TRAIL) was enhanced by DHA in human prostate cancer cells [19] and by ARS in human cervical carcinoma cells [20]. In breast cancer cells, combination treatment of DHA with doxorubicin [21] or holotransferrin [22] showed more effective antitumor activity than single drugs treatment. Combination treatment of DHA and gemcitabine exhibited strong synergistic action against pancreatic cancer cells [10] and A549 cells [11] with minimal effects on normal cells. Similar synergistic anticancer action was also observed for the combination treatment of ARS with other drugs in pancreatic cancer cells [18], osteosarcoma cells [23] and leukemia cells [24].

Activation of the Ras signaling pathway is a ubiquitous event in HCC, which contributes to the development of cancer-initiating cells and the resistance of HCC cells to apoptosis [25]. Farnesylthiosalicylic acid (FTS, salirasib), a Ras inhibitor, is an S-farnesylcysteine analog that dislodges Ras from its membrane anchorage sites and facilitates its degradation, thereby damages the down-stream signaling pathway of Ras and inhibits Ras-dependent cell growth [26,27]. FTS exhibits potent anticancer activity in many cancer cell lines *in vitro* and *in vivo* [28–31] and also shows synergistic anticancer effect with other drugs [32]. Combination treatment of FTS and gemcitabine exhibited synergistic anticancer effect in pancreatic cancer [30,33] and lung cancer [34]. Charette and coworkers [35] found that FTS sensitized HCC cells to TRAIL-induced apoptosis. The proliferation of nonsmall-cell lung carcinoma cells (A549), colon carcinoma cells and thyroid carcinoma cells can be inhibited by combination treatment of FTS and histone deacetylase inhibitor through down-regulating Ras and blocking the expression of survivin [36]. In colorectal cancer cells, combination treatment of FTS and β-catenin inhibitor PKF115-584 also showed synergistic inhibitory effect [37].

This study aims to evaluate the anticancer effect of the combination of DHA/ARS with FTS in HCC cells (Huh-7 and HepG2 cell lines). Our data demonstrated that FTS significantly enhanced the sensitivity of both Huh-7 and HepG2 cell lines to DHA/ARS by enhancing the DHA/ARS-induced Bak/Bax activation, loss of mitochondrial membrane potential (ΔΨm), release of cytochrome *c*, and caspase-8 and -9 activations. Most importantly, caspase-3 did participate in enhancing the intrinsic apoptotic pathway in a positive feedback loop fashion to mediate the cytotoxicity of the combination treatment of FTS and DHA/ARS. Furthermore, ROS was involved in the anticancer action of the combination treatment of DHA and FTS instead of the combination treatment of ARS and FTS in the two HCC cell lines.

## Materials and methods

### Materials

ARS and DHA were purchased from Holleypharm (Chongqing, China). FTS was purchased from Santa Cruz Biotechnology (California, USA). DHA, ARS and FTS solutions were prepared by dissolving them in dimethylsulphoxide (DMSO, Sigma, USA) before experiments. The final concentration of DMSO was less than 1% in all experiments. Cell Counting Kit-8 (CCK-8) was purchased from Dojindo Laboratories (Kumamoto, Japan). 2′, 7′-dichlorodihydrofluorescein diacetate (DCF-DA) and N-acetyl-cysteine (NAC) were purchased from Sigma-Aldrich (St. Louis, USA). Mito-Tracker Deep Red 633 was purchased from Invitrogen (Massachusetts, USA). Ac-DEVD-CHO and Z-VAD-FMK were purchased from Beyotime (Jiangsu, China). Z-IETD-FMK was purchased from Selleckchem (USA). Mouse monoclonal anti-Bak antibody (Ab-2) and anti-Bax antibody (6A7) were purchased from Calbiochem (San Diego, CA, USA). Rabbit monoclonal anti-Bid and mouse monoclonal anti-Tubulin, anti-Bax and anti-Bak antibody were purchased from Cell Signaling Technology (Beverly, MA, USA). Proper goat anti-mouse and goat anti-rabbit secondary antibodies were purchased from Invitrogen (Massachusetts, USA). Turbofect^™^ transfection regent was purchased from Thermo Fisher Scientific (Massachusetts, USA).

### Cell culture, transfection and treatment

Huh-7 cell line was obtained from the Department of Medicine, Jinan University (Guangzhou, China). HepG2 cell line was purchased from the Experimental Animal Center, SUN YAT-SEN University (Guangzhou, China). Both Huh-7 and HepG2 cell lines were cultured in Dulbecco’s Modified Eagle’s Medium (DMEM, Gibco, Grand Island) supplemented with 10% fetal bovine serum (FBS, Sijiqing, Hangzhou, China), 1% penicillin and streptomycin (Gibco, Grand Island). Cells were maintained in a humidified incubator with 5% CO_2_ at 37°C.

For gene transfection, cells were transfected with 0.5 μg plasmids using Turbofect^™^ transfection regent in 35-mm dish for 24 before different treatments.

Lethal concentration of 100 μM DHA/ARS for Huh-7 cell line and 40 μM DHA/ARS for HepG2 cell line were used for every experiment described here. Cells were pretreated with 80 μM of FTS for 2 h, and then co-treated with DHA or ARS for 46 h.

### Assay of cell viability and apoptosis

Cell viability was assessed by CCK-8 according to manufacturer’s protocol as previously described [12]. Results reflect the average of at least three replicates. Cell morphological changes were recorded using a digital camera (Sony, Japan) after treated with different treatments for the time indicated.

Cell apoptosis detection was performed by using flow cytometry (FCM, FACSCCanto II, BD Biosciences, New Jersey, USA) analysis using Annexin V-FITC/PI apoptosis detection kit (Bestbio, Shanghai, China) according to the manufacture's protocol as previously described [12] and 10,000 events were recorded in each sample. Results reflect the average of three replicates. Apoptotic cells were those stained with Annexin V-FITC^+^/PI^−^ (early apoptotic cells) and Annexin V-FITC^+^/PI^+^ (late apoptotic cells).

### Drug combination study

CCK-8 assay was used to assess the dose-response cytotoxicity of FTS/DHA/ARS after treatment for 48 h in both Huh-7 and HepG2 cell lines, and the IC_50_ value of each drug was calculated by using CompuSyn software [38]. Experiments of two-drug combination were designed according to the “diagonal scheme” [39]. Briefly, the dose-range of FTS/DHA/ARS used were 0, 0.25 × IC_50_, 0.5 × IC_50_, IC_50_, 2 × IC_50_ and 4 × IC_50_, and the combination ratio of FTS to DHA/ARS was 1:1 of (IC_50_)_FTS_/(IC_50_)_DHA/ARS_. Cell viability of HCC cells treated with various concentrations of single drug for 48 h or pretreated with FTS for 2 h and then co-treated with DHA or ARS for 46 h was assessed by CCK-8 assay. Five dilutions of each drug and their combination were used to generate the dose-effect curve for combination index (CI) calculation [38]. The CI value of the combination treatment of two drugs was calculated according to Eq 1 as follow by using CompuSyn software [38].

$$\text{CI}={\left(D\right)}_{1}/{\left(D_{x}\right)}_{1}+{\left(D\right)}_{2}/{\left(D_{x}\right)}_{2};$$ (1)

where (*D*_*X*_)_1_ and (*D*_*X*_)_2_ in the denominators are the doses (or concentrations) for *D*_*1*_ (drug 1) and *D*_*2*_ (drug 2) alone that gives x% inhibition, whereas (*D*)_1_ and (*D*)_2_ in the numerators are the doses of drug 1 and drug 2 in combination that also inhibits x% (i.e., isoeffective). CI < 1, CI = 1, and CI > 1 indicate synergism, additive, and antagonism effect, respectively.

### Assessment of mitochondrial membrane potential (ΔΨm)

JC-1 (Beyotime, Jiangsu, China), a mitochondrial membrane potential assay kit, was used to analyze loss of ΔΨm by using fluorescence microscopic imaging and FCM analysis. The dye molecules of J-aggregates in the mitochondria matrix and emit red fluorescence when mitochondria have high ΔΨm, and the dye molecules of JC-1 are monomer and emit green fluorescence when mitochondria have low ΔΨm [40]. After different treatments for 48 h, the cells were stained with JC-1 (5 μg/mL) at 37°C for 20 min in the dark and then washed with JC-1 staining buffer twice before detection.

Fluorescence images of cells stained with JC-1 were obtained by using a fluorescence microscope (Olympus IX73 equipped with a CCD camera, Japan). Excitation/emission wavelengths were 480/20 nm (excitation) and 530/20 nm (emission) for JC-1 monomer (green), and 545/20 nm (excitation) and 597.5/50 nm (emission) for J-aggregates (red). For FCM analysis, the excitation wavelength was 488 nm, and the emission wavelengths were 530/30 nm (to detect the monomer) and 630/22 nm (to detect the J-aggregates).

### Detection of subcellular distributions of cytochrome *c*

After transfected with GFP-cytochrome *c* (GFP-Cyt.*c*) plasmid [41] for 24 h, the cells were treated with different treatments for 12 h, then incubated with 250 nM Mito-Tracker Deep Red 633 at 37°C for 30 min in the dark to label mitochondria, and washed twice with PBS before imaging. Subcellular distributions of cytochrome *c* were visualized using a fluorescence microscope (Olympus IX73 equipped with a CCD camera, Japan). Excitation/emission wavelengths for each of the fluorescent substances were: 488/500–550 nm for GFP-Cyt.*c*, and 633/650–750 nm for Mito-Tracker Deep Red 633.

### Imaging caspase-8/9 activation in living cells

Activation of caspase-8/9 in living cells were measured by using fluorescence resonance energy transfer (FRET) plasmids FRET-Bid consisting encoded Bid (a cleave substrate of caspase-8) fused with yellow fluorescent protein (YFP, the acceptor) and cyan fluorescent protein (CFP, the donor) [42], and SCAT9 consisting the caspase-9 cleavage sequence LEHD that is fused with a variant of YFP (Venus, the acceptor) and enhanced cyan fluorescent protein (ECFP, the donor) [43], respectively. Relative activation level of caspase-8/9 in living cells expressing FRET-Bid/SCAT9 were evaluated by measuring the ratio of [donor]/[acceptor] emission intensity [42,43].

$$\text{ratio}=I_{\text{CFP}}/I_{\text{YFP}/\text{Venus}},$$ (2)

where *I*_CFP_ is the fluorescence intensity of donor channel (Emission 480/22 nm) during donor excitation (Excitation 455/25 nm) and *I*_YFP/Venus_ is the fluorescence intensity of acceptor channel (Longpass emission 530 nm) during donor excitation (Excitation 455/25 nm). A dual-channel wide-field microscopic imaging system consists of a wide-field microscope (Axio Observer, Carl Zeiss, Oberkochen, Cermany) and two CCD cameras (AxioCam MRm, Carl Zeiss, Oberkochen, Germany) was used for imaging the caspase-8/9 activation in living cells [44]. The digital fluorescence images were then processed by using Matlab software (MathWorks, USA).

### Analysis of the activations of Bak and Bax

The activations of Bak and Bax were analyzed by FCM assay as previously described [12]. Cells were seeded in six-well plates and cultured overnight to a confluence of 50–70%, and then treated with different treatments for 48 h. Cells were harvested by trypsinization and fixed with 4% formaldehyde in PBS for 30 min, then permeabilized with PBS that containing 0.1% Txiton-100 for 10 min. After being blocked with 1% bovine serum albumin for 1 h, cells were incubated with either anti-Bax (6A7) or anti-Bak (Ab-2) (1:50) antibody at 4°C overnight and then washed with PBS three times before incubated with FITC-conjugated anti-mouse secondary antibodies (1:200) at room temperature for 1 h in the dark. After washed three times with PBS, the samples were analyzed by FCM. The results of each condition were calibrated by values for cells stained with mouse IgG as the primary antibody. Values for untreated controls were normalized to 100%. In parallel, the cells for each condition were stained with antibodies to total Bax or Bak for comparison.

### Silencing of gene expression with short hairpin RNA (shRNA) expression vectors

Cells were seeded in 24-well plates and cultured overnight to reach 70–90% confluence before transfection. Cells were transfected with shRNA expression vectors using Turbofect^™^ transfection regent according to manufacturer’s recommendations. The cells were collected by trypsinization after transfection for 24 h, and processed in the following experiment. The shRNA expression vectors of Bak and Bax were purchased from GenePharma (Shanghai, China).

### Measurement of intracellular ROS generation

ROS generation was measured by FCM with an oxidation-sensitive probe DCF-DA as previously described [12]. DCF-DA can be cleave by nonspecific esterases and becomes highly fluorescent DCF upon oxidation by ROS. Cells were cultured with different treatments for 2 h, and then stained with 20 μM DCF-DA in PBS at 37°C for 30 min in the dark. After washed twice with PBS, cells were harvested. The oxidation-induced increase of DCF fluorescence was assayed by FCM subsequently.

### Imaging Ras activation in living cells

Activation of Ras in living cells expressing FRET plasmid (Raichu-Ras) consisting H-Ras, the Ras-binding domain of Raf (Raf RBD), and a pair of YFP (the acceptor) and CFP (the donor) [45] was imaged by performing PbFRET quantification on a fluorescence microscope (Olympus IX73 equipped with a CCD camera, Japan) as follow [46,47].

$$E=\frac{1-I_{DD}/I_{DD}^{post}}{1-\left(1-x/n\right)I_{DD}/I_{DD}^{post}},$$ (3)

where *E* is FRET efficiency, *n* = 1 and *x* is the degree of acceptor photobleaching,

$$x=\frac{I_{AA}-I_{AA}^{post}}{I_{AA}};$$ (4)

*I*_*DD*_ is the fluorescence intensity of donor with donor excitation (Excitation 435/20 nm, Emission 480/40 nm), *I*_*AA*_ is the fluorescence intensity of acceptor with acceptor excitation (Excitation 510/17 nm, Emission 550/40 nm); the upper ‘*post’* indicates the fluorescence intensity after partial acceptor photobleaching with the maximum acceptor excitation (Excitation 510/17 nm). The pixel-to-pixel images of *E* were processed by using the Matlab software (MathWorks, USA).

### Western blotting analysis

Cells were collected and resuspended in ice-cold whole cell lysis buffer (10 mM Tris at pH 7.4, 1 mM NaF, 1 mM Na_3_VO_4_, 1 mM PMSF, 0.1% SDS, 1% Triton X-100, plus protease inhibitor cocktail). Equal amount of total protein, quantified by using the Bradford assay, were separated by SDS-PAGE electrophoresis and transferred on to polyvinylidene fluoride (PVDF) membranes according to standard techniques. Membranes were probed with the indicated primary antibodies overnight at 4°C followed by incubation 2 h at room temperature with fluorescent secondary antibodies. Finally, the membranes were scanned using Odyssey Infrared Imaging System (LI-COR Biosciences, Nebraska, USA). Antibodies were used according to the manufacturer’s recommendations. Tubulin was used as loading control.

### Statistics

Data were presented as mean ± SD from at least three independent experiments. The Student's t test was used to evaluate the significance of difference between two groups and the One-Way ANOVA with F test was used for comparison among three or more groups. Statistical and graphic analyses were done using the software SPSS 19.0 (SPSS, Chicago) and Origin 8.5 (OriginLab Corporation). *P* < 0.05 was defined as statistically significant difference.

## Results

### Synergistic cytotoxicity of the combination treatment of DHA/ARS and FTS in HCC cells

**Fig 1:**
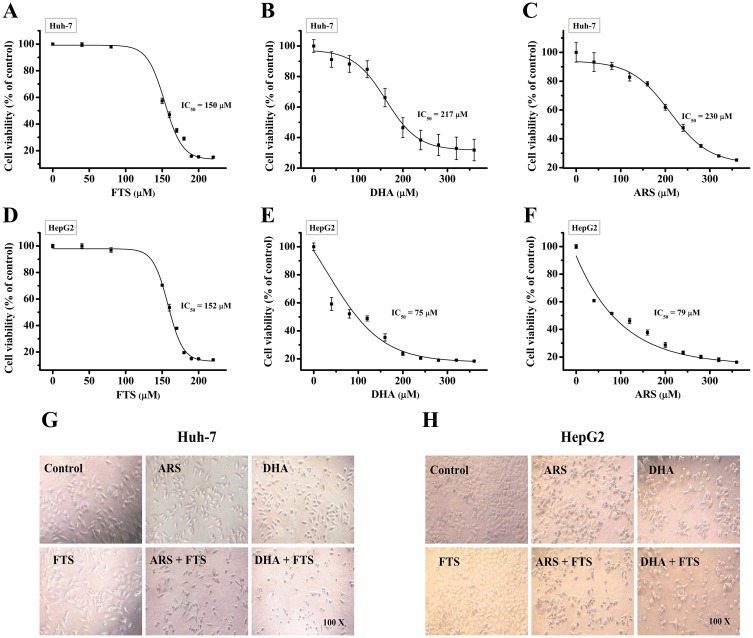
Synergistic cytotoxicity of the combination treatment of DHA/ARS and FTS in HCC cells. (**A**–**F**) Dose-response effects of FTS, DHA or ARS alone on Huh-7 cells (**A**–**C**) and HepG2 cells (**D**–**F**) growth. Cells were treated with increasing doses of FTS, DHA and ARS for 48 h and then analyzed by CCK-8 assay. (**G** and **H**) Morphological analysis on the anti-proliferation effect of DHA/ARS in the presence or absence of FTS in Huh-7 cells (**G**) and HepG2 cells (**H**). Original magnification: 100×.

We first used CCK-8 assay to evaluate the cytotoxicity of different doses of FTS/DHA/ARS in Huh-7 and HepG2 cells, and found that treatment with FTS or DHA/ARS alone induced dose-dependent cytotoxicity (Fig 1A–1F). The IC_50_ value calculated by CompuSyn software [38] was about 150 μM for FTS, 217 μM for DHA and 230 μM for ARS in Huh-7 cells, and 152 μM for FTS, 75 μM for DHA, and 79 μM for ARS in HepG2 cells (Table 1).

**Table 1:** IC_50_ of FTS/DHA/ARS in both Huh-7 and HepG2 cells.

	Huh-7			HepG2		
	FTS	DHA	ARS	FTS	DHA	ARS
IC_50_ (μM)	150	217	230	152	75	79

Synergistic cytotoxicity of two drugs was assessed according to the “diagonal scheme” [39]. As shown in Tables 2 and 3, the CI value of the combination treatment of 80 μM FTS and 100 μM DHA/ARS was 0.57/0.66 in Huh-7 cells (Table 2), and the CI value of the combination treatment of 80 μM FTS and 40 μM DHA/ARS was 0.71/0.76 in HepG2 cells (Table 3), indicating the synergistic cytotoxicity of two drugs in HCC cells. The combination treatment of 100 μM DHA/ARS and 80 μM FTS or 40 μM DHA/ARS and 80 μM FTS were used in subsequent experiments without indication. Morphological analysis also showed that FTS potently enhanced the ARS/DHA-induced cell death in both Huh-7 (Fig 1G) and HepG2 (Fig 1H) cell lines.

**Table 2:** Synergistic cytotoxicity of the combination treatment of DHA/ARS and FTS in Huh-7 cells.

FTS		DHA		ARS		DHA + FTS		ARS + FTS	
Dose (μM)	IR (%)*	Dose (μM)	IR (%)*	Dose (μM)	IR (%)*	IR (%)*	CI	IR (%)*	CI
40	0.0 ± 1.8	50	19.4 ± 1.2	50	14.6 ± 3.5	28.8 ± 1.4	0.76	18.5 ± 3.3	0.96
80	1.3 ± 3.4	100	29.9 ± 2.7	100	23.7 ± 4.5	66.0 ± 1.5	0.57	58.3 ± 3.3	0.66
160	56.6 ± 2.0	200	47.2 ± 0.6	200	53.9 ± 3.5	69.7 ± 0.8	1.04	71.1 ± 0.5	1.00
320	77.9 ± 0.6	400	81.3 ± 0.2	400	79.8 ± 0.4	83.5 ± 0.9	1.42	83.1 ± 0.3	1.46
640	82.0 ± 0.3	800	83.1 ± 0.3	800	82.8 ± 0.7	84.0 ± 0.5	2.79	84.1 ± 0.7	2.83

*IR (%): Inhibition rate (%) = 1 − (Cell viability (%))

**Table 3:** Synergistic cytotoxicity of the combination treatment of DHA/ARS and FTS in HepG2 cells.

FTS		DHA		ARS		DHA + FTS		ARS + FTS	
Dose (μM)	IR (%)*	Dose (μM)	IR (%)*	Dose (μM)	IR (%)*	IR (%)*	CI	IR (%)*	CI
40	0.9 ± 1.1	20	29.8 ± 4.6	20	25.1 ± 2.3	42.4 ± 3.1	0.62	31.2 ± 3.4	0.84
80	0.6 ± 0.5	40	41.9 ± 3.1	40	36.7 ± 2.2	56.3 ± 3.9	0.71	53.1 ± 2.1	0.76
160	38.1 ± 2.8	80	47.3 ± 1.6	80	46.9 ± 4.1	77.5 ± 0.1	0.62	78.7 ± 0.7	0.62
320	78.7 ± 0.5	160	70.0 ± 7.1	160	68.5 ± 3.1	78.7 ± 0.4	1.19	80.9 ± 0.8	1.14
640	82.0 ± 0.4	320	78.1 ± 0.4	320	80.6 ± 0.3	79.4 ± 0.2	2.29	83.8 ± 0.3	2.00

*IR (%): Inhibition rate (%) = 1 − (Cell viability (%))

### FTS enhances DHA/ARS-induced apoptosis

**Fig 2:**
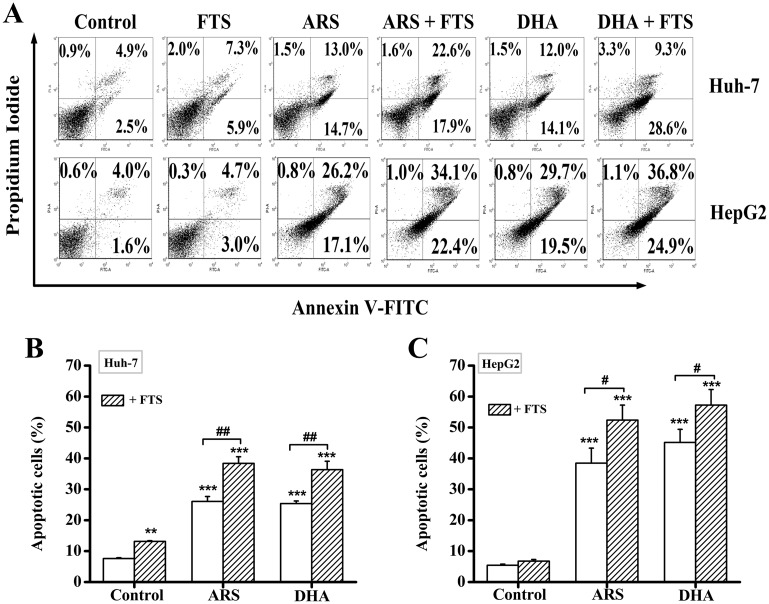
FTS enhances DHA/ARS-induced apoptosis in HCC cells. (**A**) Typical FCM analysis of apoptosis induced by DHA/ARS in the presence or absence of FTS. Cells were treated with DHA/ARS for 48 h with or without the addition of FTS and then stained with Annexin V-FITC/PI before being analyzed by FCM. (**B** and **C**) Statistical results of three independent FCM analyses on apoptosis in Huh-7 (**B**) and HepG2 (**C**) cells. **P < 0.01 and ***P < 0.001, compared with control; ^#^*P* < 0.05 and ^##^*P* < 0.01.

We next evaluated the form of cell death induced by the combination treatment of DHA/ARS and FTS by using FCM analysis with Annexin V-FITC/PI double staining. The representative dot-plots illustrating apoptotic status are shown in Fig 2A and the corresponding statistical results from three independent experiments are shown in Fig 2B and 2C. The percentage of apoptotic cells including Annexin V-FITC^+^/PI^−^ (early apoptotic cells) and Annexin V-FITC^+^/PI^+^ (late apoptotic cells) induced by the combination treatment increased from 27.7 ± 1.61% (ARS) and 26.1 ± 0.86% (DHA) to 40.5 ± 2.15% (ARS + FTS) and 37.9 ± 2.71% (DHA + FTS) in Huh-7 cells (Fig 2A and 2B). In HepG2 cells, the percentage of apoptotic cells induced by the combination treatment increased from 38.5 ± 4.85% (ARS) and 45.1 ± 4.24% (DHA) to 52.4 ± 4.85% (ARS + FTS) and 58.2 ± 5.05% (DHA + FTS) (Fig 2A and 2C). Taken together, FTS significantly enhanced DHA- or ARS-induced apoptosis in both Huh-7 and HepG2 cell lines.

### FTS promotes DHA/ARS-induced mitochondrial membrane depolarization and cytochrome *c* release

**Fig 3:**
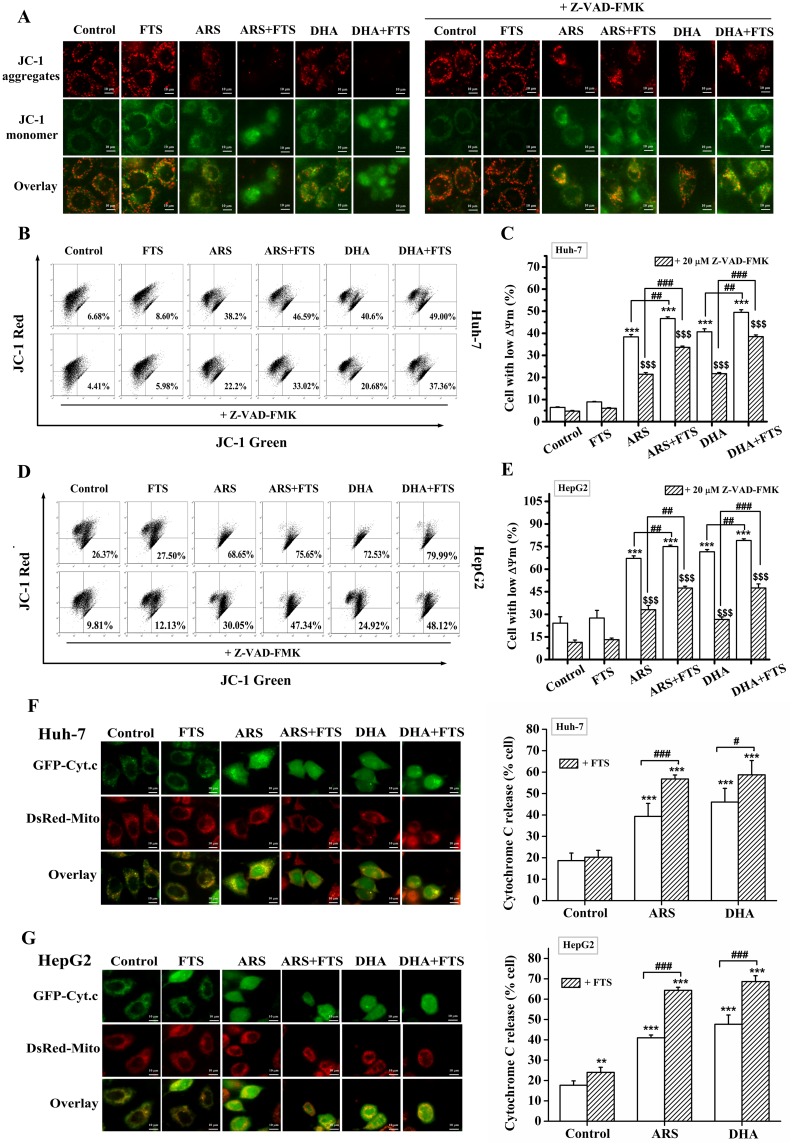
FTS promotes DHA/ARS-induced mitochondrial membrane depolarization and cytochrome *c* release. (**A**) Fluorescence images of HepG2 cells stained with JC-1 (5 μg/mL) under different treatments. (**B** and **D**) Typical FCM results on the loss of ΔΨm in Huh-7 cells (**B**) and HepG2 (**D**) cells. Cells were pretreated with Z-VAD-FMK (pan-caspase inhibitor, 20 μM) for 30 min before FTS/DHA/ARS treatment or the combination treatment of FTS and DHA/ARS for 48 h and then stained with JC-1 before being analyzed by FCM. (**C** and **E**) Statistical results of three independent FCM analyses on ΔΨm loss in Huh-7 (**C**) and HepG2 (**E**) cells. Scale Bar: 10 μm. ***P < 0.001, compared with control from the group of without Z-VAD-FMK; ^*$ $ $*^*P* < 0.001, compared with control from the group of with Z-VAD-FMK; ^*##*^*P <* 0.01 and ^*###*^*P <* 0.001. (**F** and **G**) Fluorescence images (left) of cells expressing GFP-Cyt.*c* under different treatments and statistical results (right) of cells showing cytochrome *c* release from 300 cells in three independent experiments in Huh-7 (**F**) and HepG2 (**G**) cells. Cells transfected with GFP-Cyt.*c* plasmid were under different treatments for 12 h, and then stained with Mito-tracker red to label mitochondria before fluorescence microscopic imaging. Scale bar: 10 μm. **P < 0.01 and ***P < 0.001, compared with control; ^#^*P* < 0.05 and ^###^*P* < 0.001.

Depolarization of mitochondrial membrane resulting in a loss of ΔΨm is a universal event during the intrinsic apoptotic pathway [48]. We used fluorescence microscopic imaging to assess the loss of ΔΨm by imaging the cells stained with JC-1. As shown in Fig 3A, control and FTS-treated HepG2 cells mainly exhibited red fluorescence. DHA/ARS-treated cells exhibited a green fluorescence with sporadic red fluorescence, indicating that DHA/ARS induced significant loss of ΔΨm. Furthermore, FTS pretreatment potently enhanced the green fluorescence of cells treated with DHA/ARS, indicating that FTS enhanced DHA/ARS-induced ΔΨm loss. In addition, Z-VAD-FMK (pan-caspase inhibitor) pretreatment potently prevented the reduction of red fluorescence for the cells treated with the combination treatment, demonstrating that caspases were involved in the combination treatment-induced loss of ΔΨm.

In addition, we also used FCM analysis to evaluate the loss of ΔΨm by measuring the fluorescence of JC-1. Representative FCM results are shown in Fig 3B and 3D, and the corresponding statistical results from three independent experiments are shown in Fig 3C and 3E. ARS/DHA treatment induced significant loss of ΔΨm, and FTS treatment did not induce significant ΔΨm loss, but FTS pretreatment significantly enhanced the ARS/DHA-induced ΔΨm loss in the presence or absence of Z-VAD-FMK pretreatment in both Huh-7 (Fig 3B and 3C) and HepG2 (Fig 3D and 3E) cell lines. Moreover, Z-VAD-FMK pretreatment significantly inhibited the loss of ΔΨm induced by various stimuli in both Huh-7 (Fig 3B and 3C) and HepG2 (Fig 3D and 3E) cell lines, indicating the important role of caspases in regulating the loss of ΔΨm in the two cell lines.

We next investigated the distribution of cytochrome *c* in single living cells expressing GFP-Cyt.*c* by using a fluorescence microscope. As shown in Fig 3F and 3G, GFP-Cyt.*c* completely localized on mitochondria (DsRed-Mito) in control cell, FTS treatment did not induce GFP-Cyt.*c* release from mitochondria in Huh-7 cells (Fig 3F) but induced a modest but significant GFP-Cyt.*c* release from mitochondria in HepG2 cells (Fig 3G). Furthermore, FTS pretreatment significantly enhanced the DHA/ARS-induced GFP-Cyt.*c* release from mitochondria in the two cell lines (Fig 3F and 3G). Statistical results from 300 cells in three independent experiments (Fig 3F) showed that the percentage of cells showing cytochrome *c* release induced by the combination treatment increased from 39.3 ± 6.05% (ARS) and 46 ± 6.4% (DHA) to 56.8 ± 1.87% (ARS + FTS) and 58.7 ± 6.67% (DHA + FTS) in Huh-7 cells. In HepG2 cells, the combination treatment increased the percentage of cells with cytochrome *c* release from 41 ± 1.39% (ARS) and 47.67 ± 4.56% (DHA) to 64.33 ± 1.5% (ARS + FTS) and 68.67 ± 2.86% (DHA + FTS) (Fig 3G).

Collectively, these results demonstrated that FTS significantly promoted DHA/ARS-induced mitochondrial membrane depolarization and cytochrome *c* release in HCC cells.

### FTS promotes DHA/ARS-induced caspase-8 and -9 activations

**Fig 4:**
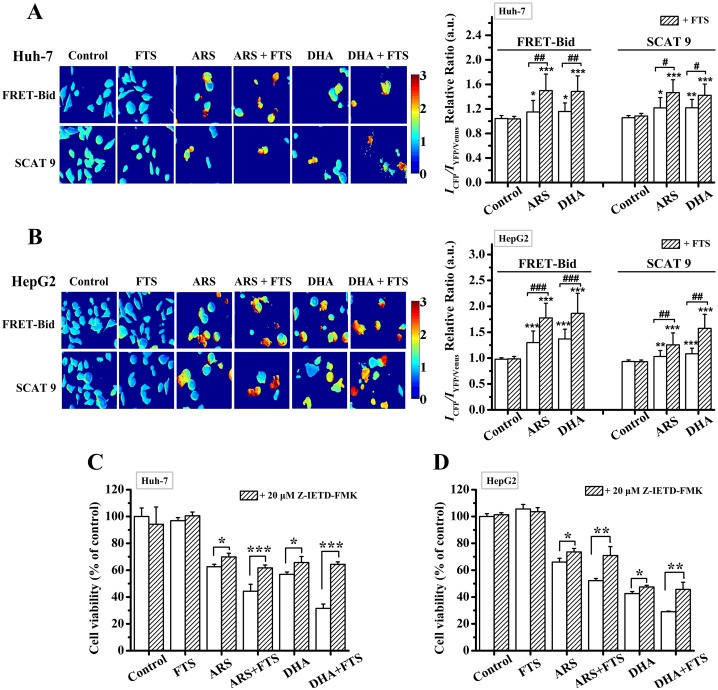
FTS promotes DHA/ARS-induced caspase-8 and -9 activations. (**A** and **B**) *I*_CFP_*/I*_YFP/Venus_ ratio images of cells expressing FRET-Bid or SCAT9 (left) and corresponding statistical results (right) from at least 250 cells in Huh-7 (**A**) and HepG2 (**B**) cells. Cells were transfected with FRET-Bid and SCAT9 plasmid for 24 h respectively and then treated with DHA/ARS for 24 h in the presence or absence of FTS before fluorescence microscopic imaging. *P < 0.05 and ***P < 0.001, compared with control; ^#^*P* < 0.05, ^##^*P* < 0.01 and ^###^*P* < 0.001. (**C** and **D**) Cells were pretreated with Z-ITED-FMK (caspase-8 inhibitor, 20 μM) for 30 min before FTS/DHA/ARS treatment or the combination treatment of DHA/ARS and FTS for 48 h and then analyzed by CCK-8 assay. *P < 0.05, **P < 0.01, and ***P < 0.001.

The ratio of [donor]/[acceptor] emission intensity (*I*_CFP_/*I*_YFP/Venus_) of living cells expressing FRET-Bid/SCAT9 [42,43] was used to evaluate the relative activation level of caspase-8/9. The *I*_CFP_/*I*_YFP/Venus_ ratio images of cells expressing FRET-Bid or SCAT9 (left) and the corresponding statistical results from at least 250 cells (right) are shown in Fig 4. FTS pretreatment significantly enhanced the DHA/ARS-induced increase in the *I*_CFP_/*I*_YFP/Venus_ ratio of cells expressing FRET-Bid and SCAT9 respectively (Fig 4), further demonstrating that FTS significantly promoted DHA/ARS-induced caspase-8 and -9 activations and subsequent apoptosis in HCC cells.

To further evaluate the role of caspase-8 in inducing cell death by the combination treatment of ARS/DHA and FTS, we next used CCK-8 assay to assess the effects of pretreatment with 20 μM Z-IETD-FMK (caspase-8 inhibitor) on the cytotoxicity of the combination treatment of DHA/ARS and FTS, and found that Z-IETD-FMK pretreatment significantly but modestly inhibited the cytotoxicity of DHA/ARS treatment in both Huh-7 (Fig 4C) and HepG2 (Fig 4D) cell lines, suggesting the modest role of caspase-8 in ARS/DHA-induced cytotoxicity. However, pretreatment with Z-IETD-FMK potently prevented the cytotoxicity of the combination treatment of ARS/DHA and FTS demonstrating the important role of caspase-8 in the combination treatment-induced cell death.

### Caspase-8 is not activated by caspase-3

**Fig 5:**
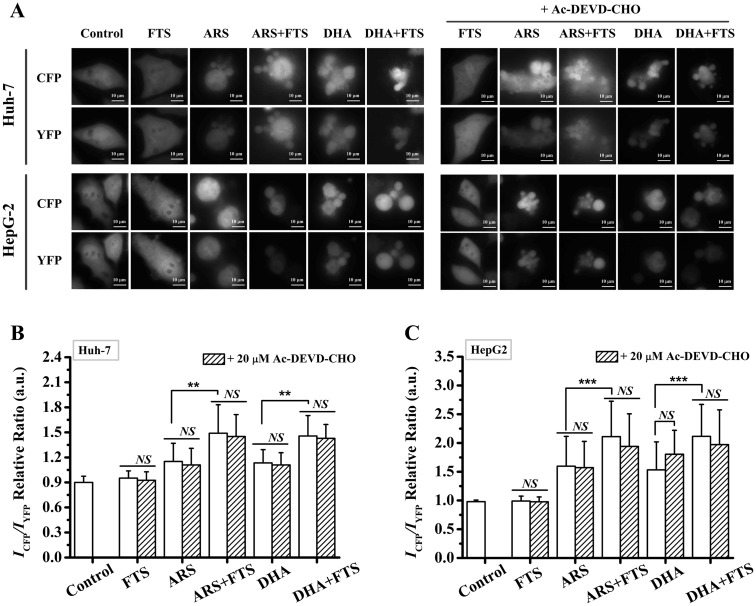
Caspase-8 is not activated by caspase-3. (**A**) Microscopic images of cells expressing FRET-Bid in the absence or presence of Ac-DEVD-CHO (caspase-3 inhibitor, 20 μM) in Huh-7 and HepG2 cells. (**B** and **C**) Statistical results of *I*_*CFP*_*/I*_*YFP*_ ratio from at least 80 Huh-7 cells (**B**) or HepG2 cells (**C**). Cells were transfected with FRET-Bid plasmid for 24 h and then pretreated with Ac-DEVD-CHO for 30 min before FTS/DHA/ARS treatment or the combination treatment of FTS and DHA/ARS for 24 h before fluorescence microscopic imaging. Scale bar: 10 μm. *NS* = no statistical significance, *P >* 0.05; **P < 0.01, ***P < 0.001.

In order to examine whether caspase-8 was activated by caspase-3, we used FRET-Bid plasmid to measure the caspase-8 activation level after different treatments in the absence or presence of 20 μM Ac-DEVD-CHO (caspase-3 inhibitor) in both Huh-7 and HepG2 cell lines (Fig 5). Although ARS/DHA treatment or the combination treatment significantly induced cell death (Fig 5A), pre-treatment with Ac-DEVD-CHO did not reduce the *I*_*CFP*_*/I*_*YFP*_ ratio of the cells treated with ARS/DHA or the combined FTS and ARS/DHA (Fig 5B and 5C), indicating that caspase-8 was not activated by caspase-3 after the combination treatment of FTS and DHA/ARS.

### Bak/Bax plays a modest but significant role in inducing apoptosis by the combination treatment

**Fig 6:**
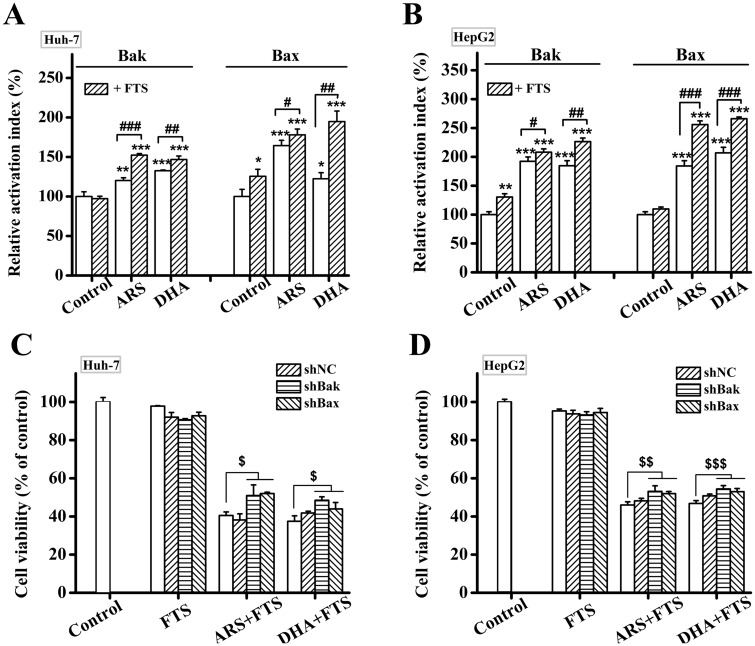
Bak/Bax plays a modest but significant role in inducing apoptosis by the combination treatment. (**A** and **B**) Combination treatment of DHA/ARS and FTS enhanced the activation of Bak/Bax compared with single drugs treatment in both Huh-7 (**A**) and HepG2 (**B**) cell lines. Cells were treated with DHA/ARS for 48 h in the presence or absence of FTS and then incubated with 6A7 monoclonal anti-Bax antibody or Ab-2 monoclonal anti-Bak antibody before being analyzed by FCM. (**C** and **D**) Effects of silencing Bak or Bax on the cytotoxicity of the combination treatment assessed by CCK-8 assays in both Huh-7 (**C**) and HepG2 (**D**) cell lines. Cells were transfected with shBak and shBax expression vectors respectively before treatment with DHA/ARS for 48 h in case of FTS pretreatment. Cells with shNC were used as negative control. *P < 0.05, **P < 0.01 and ***P < 0.001, compared with control; ^*#*^*P* < 0.05, ^*##*^*P* < 0.01 and ^*###*^*P* < 0.001; ^*$*^*P* < 0.05, ^*$ $*^*P* < 0.01 and ^*$ $ $*^*P* < 0.001.

Bak and Bax, two key pro-apoptosis proteins, are responsible for the permeabilization of the mitochondrial outer membrane [49]. Activation of Bak/Bax experiences an N-terminal conformational change, which can be measured by specific antibodies anti-Bak (Ab-2) and anti-Bax (6A7) [50]. In order to confirm the function of Bak/Bax, we firstly used FCM analysis to investigate the percentage of cells with activated Bak or Bax. The combination treatment of DHA/ARS and FTS significantly enhanced the activations of both Bak and Bax compared with single drugs treatment in both Huh-7 (Fig 6A) and HepG2 (Fig 6B) cell lines. We next silenced the gene of Bak/Bax using shRNA expression vectors to further investigate the roles of Bak/Bax in the combination treatment-induced apoptosis in HCC cells and the efficiency of gene silencing of Bak/Bax was shown in S2 Fig. CCK-8 assay showed that silencing either Bak or Bax modestly but significantly prevented the cytotoxicity of the combination treatment in both Huh-7 cells (Fig 6C) and HepG2 cells (Fig 6D). These data demonstrated that both Bak and Bax played a modest but significant role in inducing apoptosis by the combination treatment of DHA/ARS and FTS in both Huh-7 and HepG2 cell lines.

### ROS is involved in the action of the combination treatment of DHA and FTS in inducing apoptosis

**Fig 7:**
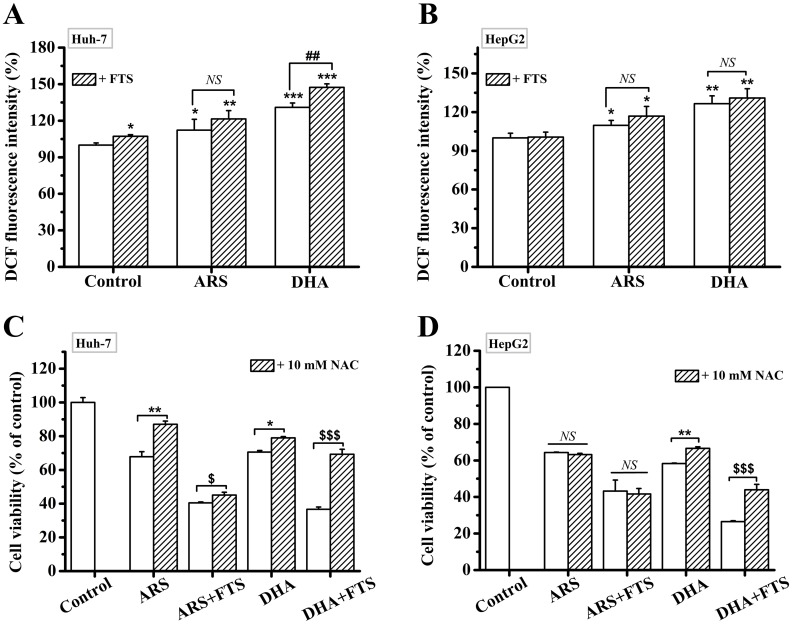
ROS is involved in the anticancer action of the combination treatment of DHA and FTS. (**A** and **B**) FTS significantly increased DHA-induced ROS generation but did not affect the ROS generation induced by ARS in Huh-7 cells (**A**), and FTS did not affect the ROS generation induced by DHA/ARS in HepG2 cells (**B**). Cells were treated with DHA/ARS for 2 h in the presence or absence of FTS and then incubated with DCF-DA before being analyzed by FCM. *NS* = no statistical significance, *P* > 0.05; *P < 0.05, **P < 0.01 and ***P < 0.001, compared with control; ^*##*^*P* < 0.01. (**C** and **D**) Effects of NAC on the cytotoxicity of the combination treatment of DHA/ARS and FTS assessed by CCK-8 assays in both Huh-7 (**C**) and HepG2 (**D**) cells. Cells were pretreated with 10 mM NAC for 2 h or not, and then treated with DHA/ARS for 48 h in the presence or absence of FTS. *NS* = no statistical significance, *P* > 0.05; *P < 0.05 and **P < 0.01; ^$ $ $^*P* < 0.001; ^*##*^*P* < 0.01 and ^*###*^*P* < 0.001.

Our recent publications have indicated that both DHA and ARS significantly induced the generation of ROS in HCC cells [12,51,52]. We here assessed intracellular ROS production at 2 h after different treatments using DCF-DA assay. In Huh-7 cells, FTS pretreatment significantly enhanced DHA-induced ROS production, while FTS did not affect ARS-induced ROS generation (Fig 7A). However, FTS pretreatment did not enhance DHA/ARS-induced ROS generation in HepG2 cells (Fig 7B). In order to confirm the function of ROS, cells were pre-incubated with 10 mM NAC, an ROS scavenger [15], for 2 h before different treatments. CCK-8 assay showed that NAC pretreatment significantly inhibited the cytotoxicity of the combination treatment of DHA and FTS compared with DHA treatment, but didn’t prevent the cytotoxicity of the combination treatment of ARS and FTS compared with ARS treatment (Fig 7C and 7D), indicating that ROS was involved in the action of the combination treatment of DHA and FTS in inducing apoptosis in both cell lines but did not participate in the action of the combination treatment of ARS and FTS.

### DHA/ARS inhibits Ras activation

**Fig 8:**
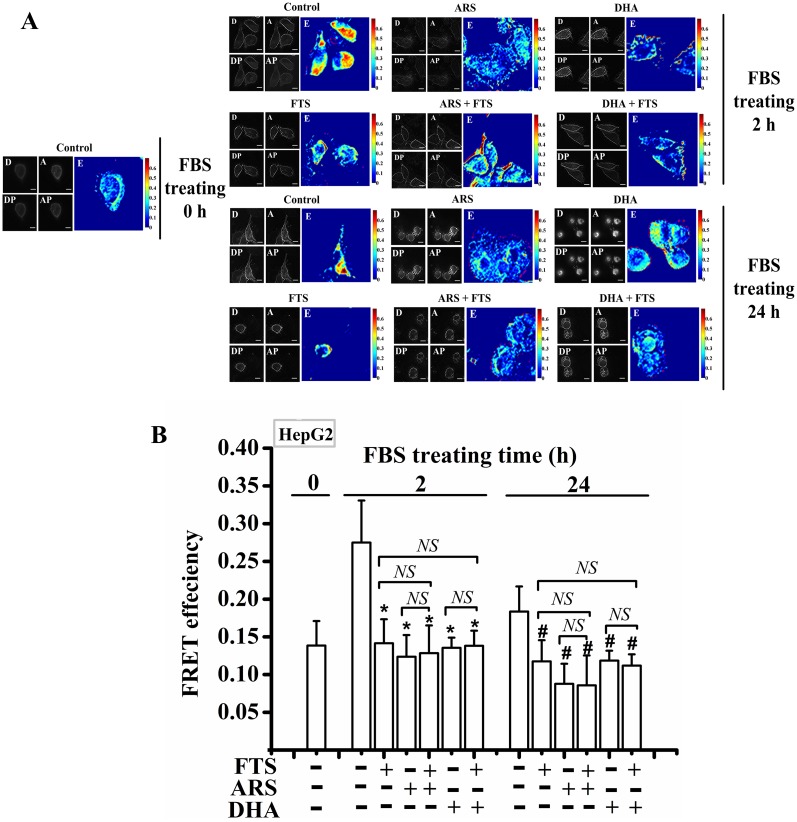
DHA/ARS inhibits Ras activation. (**A**) FRET efficiency images of HepG2 cells expressing Raichu-Ras plasmid quantified by PbFRET quantification method. D: fluorescence intensities images from donor channel (Emission 480/40 nm) during donor excitation (Excitation 435/20 nm); A: fluorescence intensities images from acceptor channel (Emission 550/40 nm) during acceptor excitation (Excitation 510/17 nm); DP: fluorescence intensities images from donor channel during donor excitation after photobleaching (Excitation 510/17 nm); AP: fluorescence intensities images from acceptor channel during acceptor excitation after photobleaching (Excitation 510/17 nm). Scale bar: 10 μm. (**B**) Statistical results of FRET efficiency from at least 90 living HepG2 cells expressing Raichu-Ras plasmid after different treatments. *NS* = no statistical significance, *P* > 0.05; *P < 0.05, compared with control from the group treated with FBS for 2 h; ^#^*P* < 0.05, compared with control from the group treated with FBS for 24 h.

It was reported that FTS strongly inhibited the Ras activation in HCC cells [31] and DHA inhibited the Ras pathway, a cell proliferation signal pathway, in HCC cells [53]. We thus used FRET imaging to examine the Ras activation levels in living HepG2 cells. HepG2 cells expressing Raichu-Ras plasmid, a FRET-based plasmid [45], were starved for 24 h, and then cultured in DMEM containing 10% FBS for 0, 2 and 24 h with different treatments, respectively. Ras activation levels in living cells were assessed by imaging the FRET efficiency between CFP and YFP using PbFRET quantification method [46,47]. The origin fluorescence intensity images from different channels and the corresponding pixel-to-pixel FRET images of representative cells expressing Raichu-Ras are shown in Fig 8A, and the statistical results from at least 90 cells are shown in Fig 8B. The FRET efficiency (E) of Raichu-Ras in living cells is proportional to the Ras activation levels. As shown in Fig 8B, FBS culture for 2 h significantly increased the level of Ras activation, and similar to FTS, ARS/DHA significantly prevented FBS-induced Ras activation, indicating the potent inhibitory effect of ARS/DHA on cell proliferation. Moreover, FTS pretreatment did not enhance the inhibitory effect of DHA/ARS on Ras activation (Fig 8B).

## Discussion

Although recent studies have indicated the potential anticancer activity of ARTs, treatment with ARTs alone does not show enough effectiveness for use in cancer treatment. The best clinical use of these compounds seems to be in combination with traditional chemotherapeutic agents to achieve a synergistic effect with fewer side effects and overcome drugs resistance [54]. To our knowledge, this report for the first time assessed the effect of FTS on the cytotoxicity of DHA/ARS in HCC cells. Our results showed that FTS significantly sensitized HCC cells to DHA/ARS by enhancing the extrinsic and the intrinsic apoptotic pathways, exhibiting a synergistic anticancer effect.

Our data that FTS significantly enhanced the DHA/ARS-induced caspase-8 and -9 activations (Fig 4A and 4B) suggest the involvement of both extrinsic and intrinsic apoptotic pathways in the action of the combination treatment. The significant inhibitory effect of Z-IETD-FMK pretreatment on the cytotoxicity of the combination treatment of DHA/ARS and FTS (Fig 4C and 4D) further demonstrate the involvement of the extrinsic apoptotic pathway. The facts that FTS significantly enhanced DHA/ARS-induced ΔΨm loss and cytochrome *c* release (Fig 3) further demonstrate the involvement of the intrinsic apoptotic pathway. Although western blotting (S1 Fig) suggested that endogenous Bid might play little role in inducing cell death by the combination treatment of DHA/ARS and FTS in HCC cells, FRET results showed that FTS significantly enhanced the DHA/ARS-induced cleavage of exogenous Bid (Fig 4A and 4B). Transfection with FRET-Bid plasmid resulted in an overexpression of exogenous Bid, which might enlarge the cleavage effect of Bid by ARS/DHA or the combination of ARS/DHA and FTS. In addition, ultrasensitivity of FRET sensor makes it possible to detect a small quantity of Bid cleavage in single living cells. We recently found that DHA/ARS significantly induced both caspase-8 and -9 activations in HCC cells [12,51,52]. Although FTS had no effect on the activity of both capase-8 and -9, it significantly increased the DHA/ARS-induced caspase-8 and -9 activations (Fig 4A and 4B), similar to the action of the combination treatment of TRAIL and FTS in HCC cells, in which FTS increased death reporter (DR) 5 expression and inhibited survivin expression to increase the caspase-8 and -9 activations level induced by TRAIL in HCC cells [35]. Similarly, FTS may inhibit survivin expression in HCC cells and thus makes HCC cells sensitive to DHA/ARS.

Our observations that silencing Bak/Bax modestly but significantly prevented the cytotoxicity of the combination treatment of DHA/ARS and FTS (Fig 6C and 6D) demonstrate the modest role of both Bak and Bax in inducing apoptosis by the combination treatment. DHA/ARS significantly induced activation of both Bak and Bax in HCC cells, consistent with our previous studies [12,51,52], while FTS significantly induced Bax activation in Huh-7 cells and Bak activation in HepG2 cells (Fig 6A and 6B). Moreover, the combination treatment significantly enhanced the activation of both Bak and Bax compared with single drugs treatment in HCC cells (Fig 6A and 6B), suggesting that FTS pretreatment potentiated the DHA/ARS-triggered activation of both Bak and Bax, which has nothing to do with the ability of FTS to activate Bak or Bax. Our previous studies showed that Bax but not Bak dominated ARS-induced intrinsic apoptotic pathway in both Huh-7 [52] and HepG2 [51] cell lines, and Bak but not Bax dominated DHA-induced intrinsic apoptotic pathway in the two cell lines [12]. However, the facts that silencing each of Bak and Bax modestly but significantly prevented the cytotoxicity of the combination treatment compared with single drugs treatment in HCC cells (Fig 6C and 6D) indicated that FTS pretreatment potentiated the DHA/ARS-triggered Bak/Bax activation and subsequent intrinsic apoptotic pathway, which were further verified by the enhanced effect of FTS on the DHA/ARS-induced ΔΨm loss and cytochrome *c* release (Fig 3). In addition, participation of both Bak and Bax in the intrinsic apoptotic pathway was one of the explanations about the better anticancer effect of the combination treatment compared with single drugs treatment.

Interestingly, our observations that Z-VAD-FMK pretreatment significantly inhibited the loss of ΔΨm induced by various stimuli in both Huh-7 (Fig 3B and 3C) and HepG2 (Fig 3A, 3D and 3E) cell lines indicate the important role of caspases in regulating the intrinsic apoptotic pathway in the two cell lines. Z-VAD-FMK pretreatment also significantly inhibited the loss of ΔΨm for the control cells, indicating that the basal activated caspases were involved in the intrinsic apoptosis pathway in the two cell lines. Some previous studies reported that caspase-3 could enhance the intrinsic apoptotic pathway in a positive feedback loop fashion in several cell lines, such as MCF-7 cells [55,56], DU145 prostate cancer cells [57,58], Hela cells [59], T leukemia cells [60] and other’s cell lines [61–63]. It was reported that caspase-3 enhanced the loss of ΔΨm and Cyt.*c* release by activating Bid/Bak protein in a positive feedback loop fashion [55,57,58,61–63]. In addition, caspase-3 could directly activate caspase-8 to enhance the intrinsic apoptotic pathway [56,58–60]. Interestingly, our recent study [64] found that although caspase-8 could be directly activated by caspase-3, the intrinsic pathway was not enhanced by caspase-3 during artemisinin-induced apoptosis in non-small cell lung cancer cells (A549 cells). In this study, our results showed that caspase-8 was not activated by caspase-3 after DHA/ARS treatment alone or the combination treatment of DHA/ARS and FTS in HCC cells (Fig 5). Therefore, we speculate that caspase-3 enhance the intrinsic apoptotic pathway by activating the upstream mediators of mitochondria in a positive feedback loop fashion during DHA/ARS- or the combination treatment-induced apoptosis in HCC cells, and the exact molecular mechanism needs to be further studied.

The facts that NAC pretreatment does not inhibit the cytotoxicity of the combination treatment of ARS and FTS compared with ARS treatment (Fig 7C and 7D) suggest that ROS is not involved in the action of the combination treatment of ARS and FTS. Our data that NAC pretreatment significantly prevents ARS-induced cytotoxicity in Huh-7 cells instead of HepG2 cells (Fig 7C and 7D) further demonstrate our recent findings that ARS induces ROS-dependent apoptosis in Huh-7 cells [52] but induces ROS-independent apoptosis in HepG2 cells [51]. FTS did not significantly enhance ARS-induced ROS generation in both Huh-7 and HepG2 cell lines (Fig 7A and 7B). It is thus reasonable that ROS does not participate in the cytotoxicity of the combination treatment of ARS and FTS in the two cell lines.

However, ROS plays a key role in the cytotoxicity of the combination treatment of DHA and FTS in HCC cells. FTS significantly enhances DHA-induced ROS generation in Huh-7 cells (Fig 7A), thus it is reasonable that ROS participates in the cytotoxicity of the combination treatment of DHA and FTS in this cell lines (Fig 7C). Interestingly, although FTS did not significantly enhance DHA-induced ROS generation in HepG2 cells (Fig 7A), NAC pretreatment did significantly prevent the cytotoxicity of the combination treatment of DHA and FTS, indicating the involvement of ROS, in this cell lines (Fig 7D). It was reported that FTS treatment arrested most of cells (68%) in G0/G1 phase in HepG2 cells [31]. Moreover, the cells arrested in G0/G1 phase are particularly sensitive to DHA treatment [65], in which ROS elicited from DHA plays a key role [66]. In contrast to ARS, DHA induced ROS-dependent apoptosis in HepG2 cells (Fig 7D). Therefore, It was reasonable that ROS was involved in the action of the combination treatment of DHA and FTS (Fig 7D) even the combination treatment did not induce more ROS generation than DHA treatment (Fig 7B) in HepG2 cells.

Similar to FTS, DHA/ARS can also significantly inhibit Ras activation (Fig 8B), suggesting that DHA/ARS induces cytotoxicity likely via inhibiting cell proliferation and inducing apoptosis in HCC cells. It was reported that ARTs significantly inhibited the Ras signaling pathway in many kinds of tumor cells [19,53,67]. The Ras pathway is frequently activated and contributes to the proliferation of cancer-initiating cells [68–70]. A number of studies from our and other groups demonstrate that DHA/ARS can significantly induce apoptosis in various cancer cells [12,17,51,52,71–73]. In addition, although each of FTS and DHA/ARS significantly inhibited Ras activation (Fig 8B), FTS did not enhance the DHA/ARS-induced inhibition of Ras activation (Fig 8B), which may be due to the complete inhibition of Ras activation by each of FTS and DHA/ARS (Fig 8B). Therefore, the combination treatment of DHA/ARS and FTS can also induce cytotoxicity by inhibiting cell proliferation and inducing apoptosis in HCC cells.

In conclusion, our results for the first time demonstrate that the combined treatment of FTS and DHA/ARS exhibits a synergistic anticancer effect in HCC cells. FTS sensitizes HCC cells to DHA/ARS by enhancing the intrinsic and extrinsic apoptotic pathways. In the two HCC cell lines, caspase-3 does not activate caspase-8, but does potently enhance the intrinsic apoptotic pathway in a positive feedback loop fashion during ARS/DHA treatment or the combination treatment. Furthermore, ROS plays a key role in inducing apoptosis by the combination treatment of DHA and FTS instead of the combination treatment of ARS and FTS in HCC cells. Our results provide a rationale for the use of the combination of ARTs and FTS in the setting of HCC in clinical practice. Further investigations are needed to explore the exact molecular mechanism by which the combination treatment induces apoptosis and the *in vivo* anticancer effect on xenograft tumors in mouse.

## Supporting information

S1 Fig Western blotting analysis on the cleavage of endogenous Bid. Soluble protein extracts obtained from Huh-7 (**A**) and HepG2 (**B**) cells after different treatment for 24 h was analyzed by western blotting with anti-Bid and anti-Tubulin antibodies, respectively.
(TIF)

S2 Fig Western blotting analysis on the effects of shBak and shBax on the expression of Bak and Bax in HCC cells. Soluble protein extracts obtained from cells that transfected with shBak or shBax expression vectors for 48 h were analyzed by western blotting with anti-Bax, anti-Bak and anti-Tubulin antibodies, respectively.
(TIF)
