# Supplementary figures and images for: Farnesylthiosalicylic acid sensitizes hepatocarcinoma cells to artemisinin derivatives
Source: PLoS One. 2017 Feb 9;12(2):e0171840. doi: 10.1371/journal.pone.0171840 (PMC5300221; doi:10.1371/journal.pone.0171840)

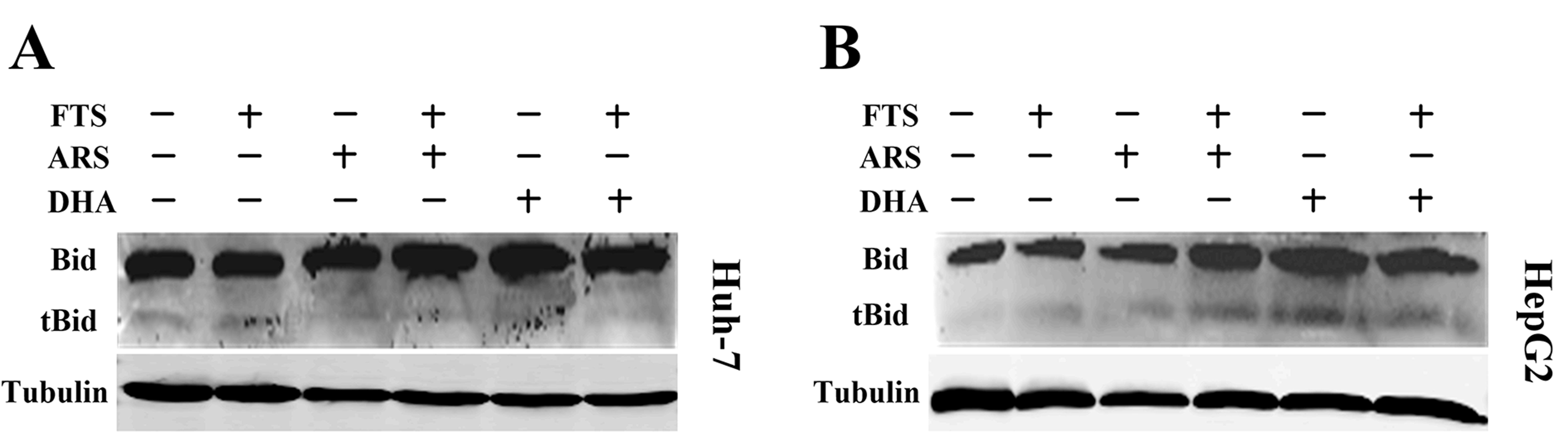

Supplement: S1 Fig — Soluble protein extracts obtained from Huh-7 (A) and HepG2 (B) cells after different treatment for 24 h was analyzed by western blotting with anti-Bid and anti-Tubulin antibodies, respectively. (TIF) [file pone.0171840.s001.tif]

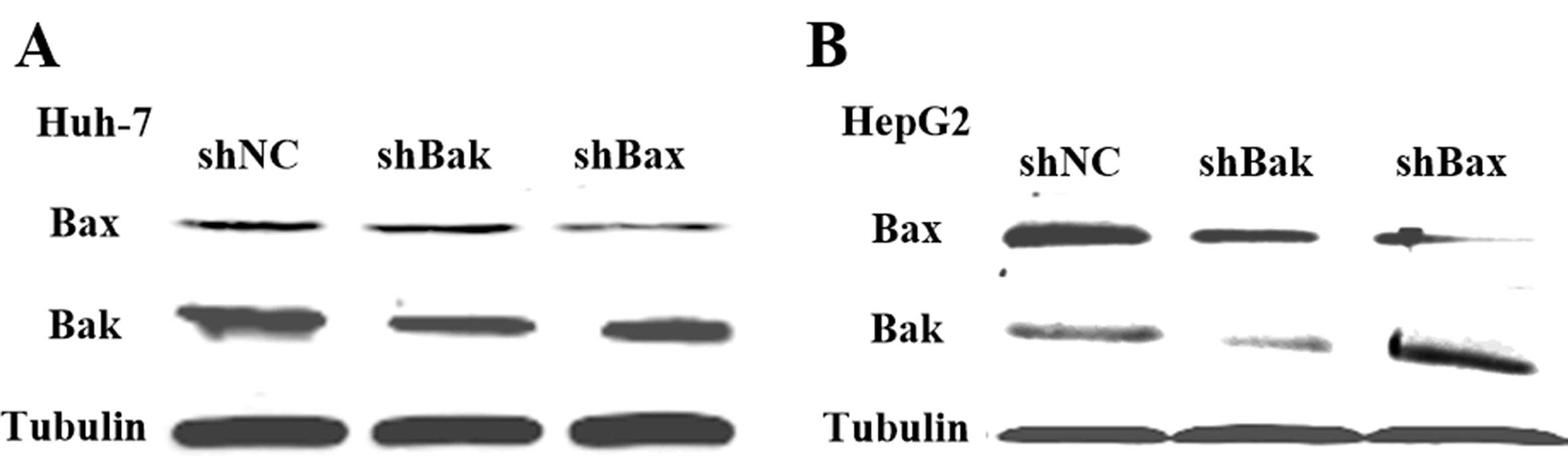

Supplement: S2 Fig — Soluble protein extracts obtained from cells that transfected with shBak or shBax expression vectors for 48 h were analyzed by western blotting with anti-Bax, anti-Bak and anti-Tubulin antibodies, respectively. (TIF) [file pone.0171840.s002.tif]
